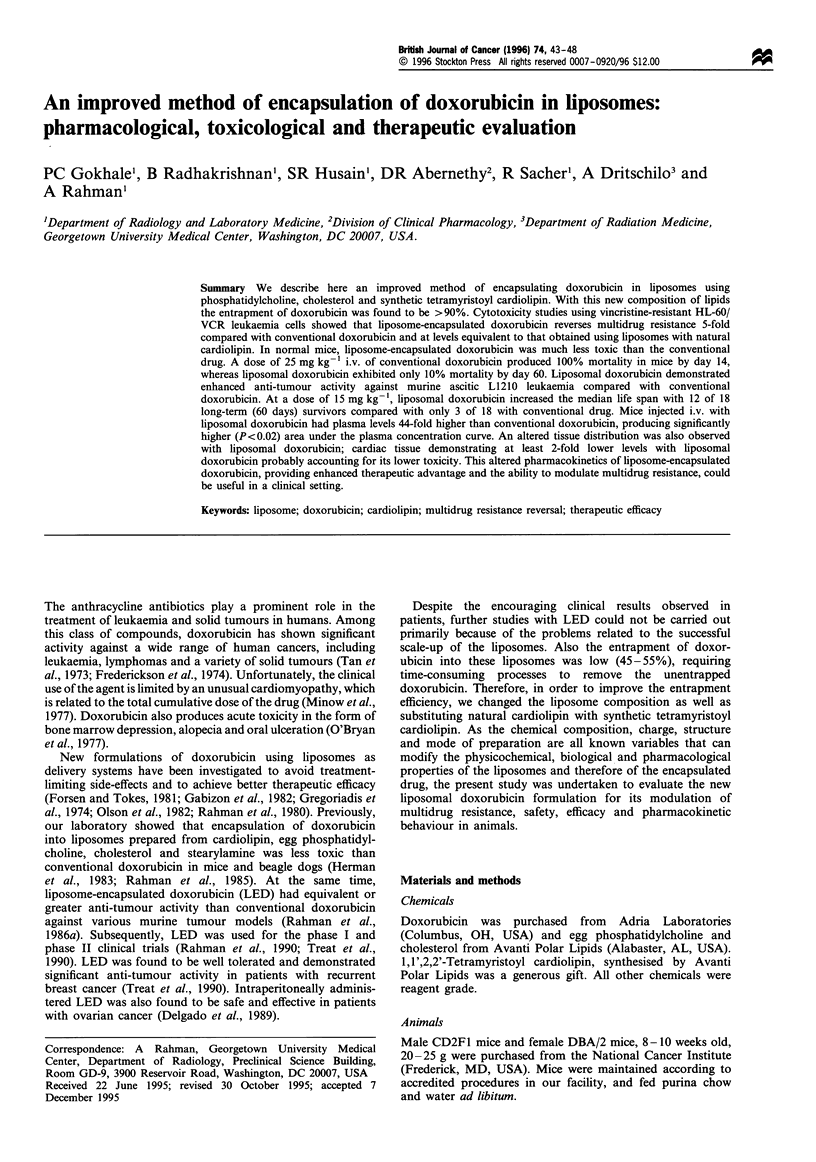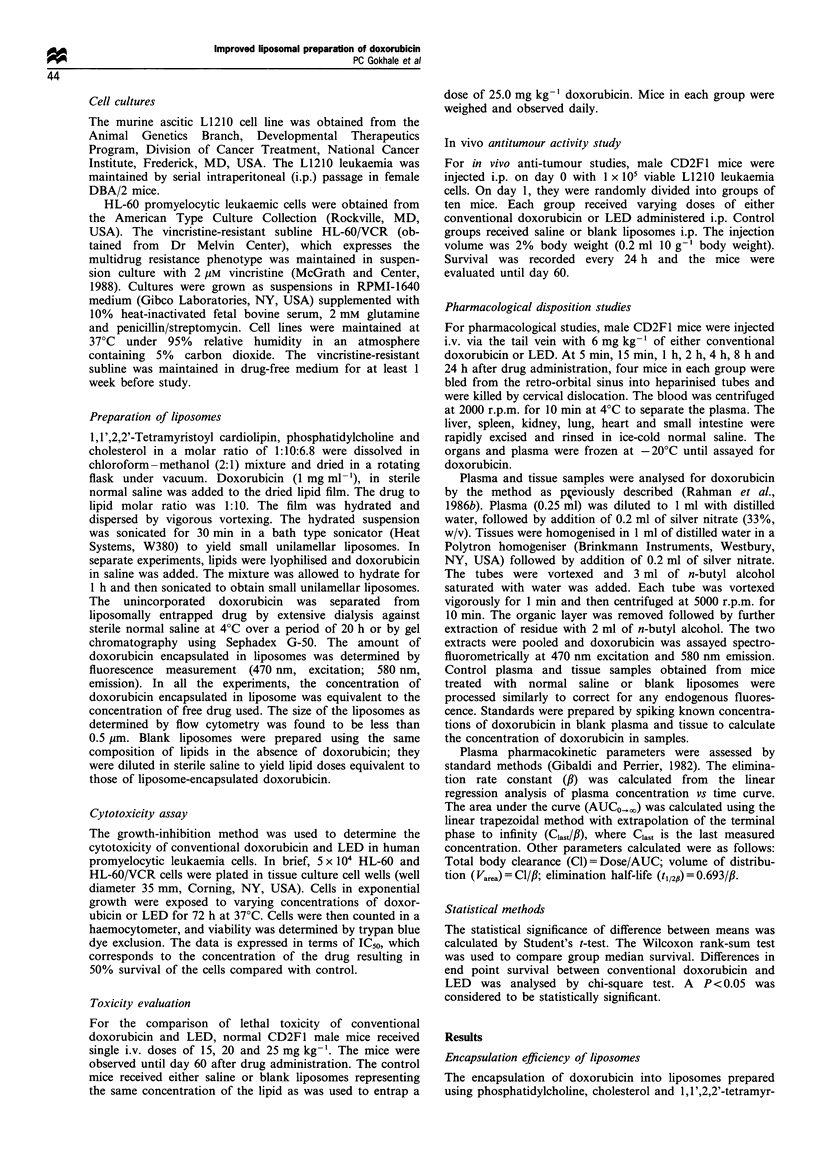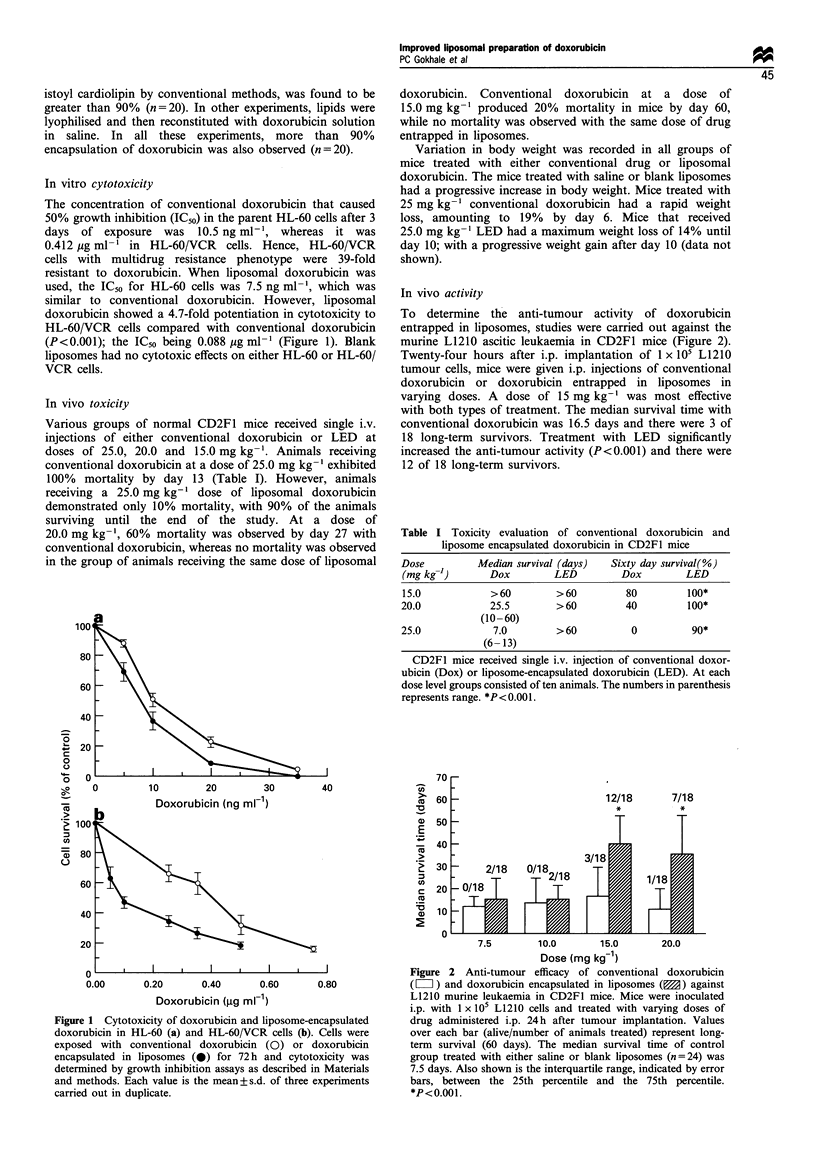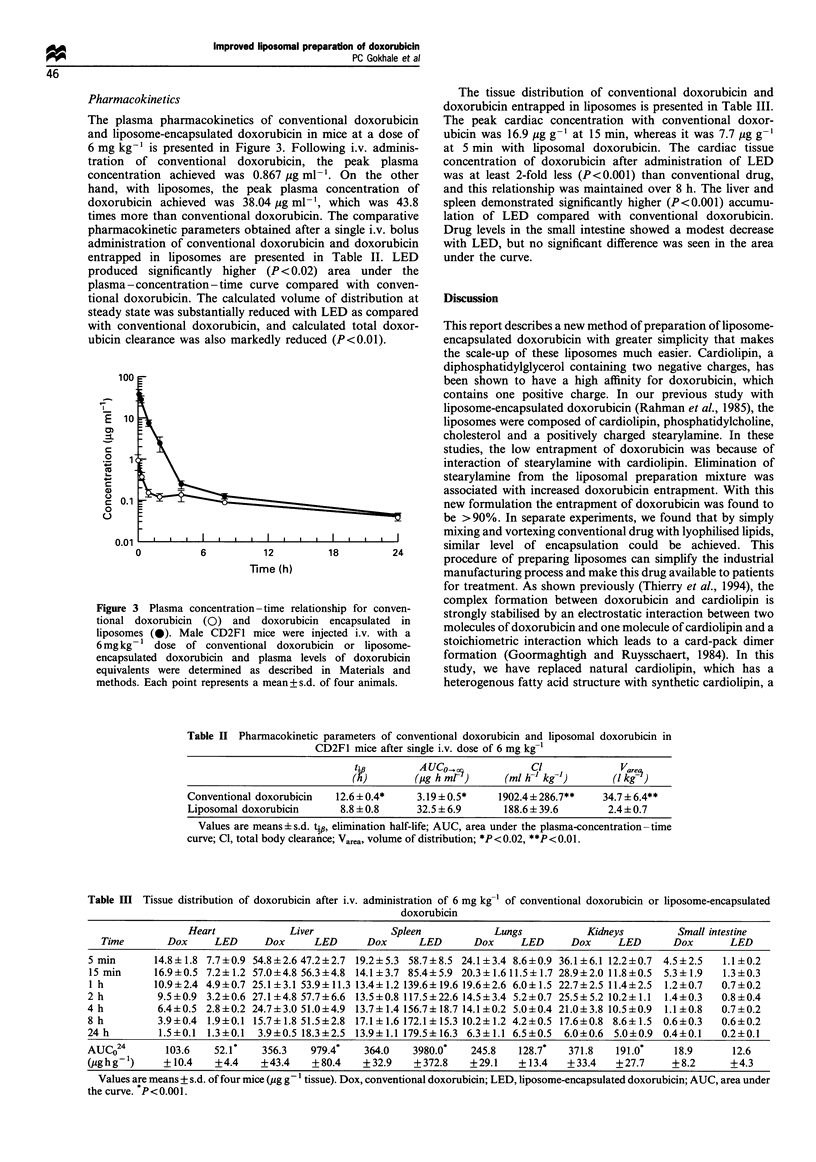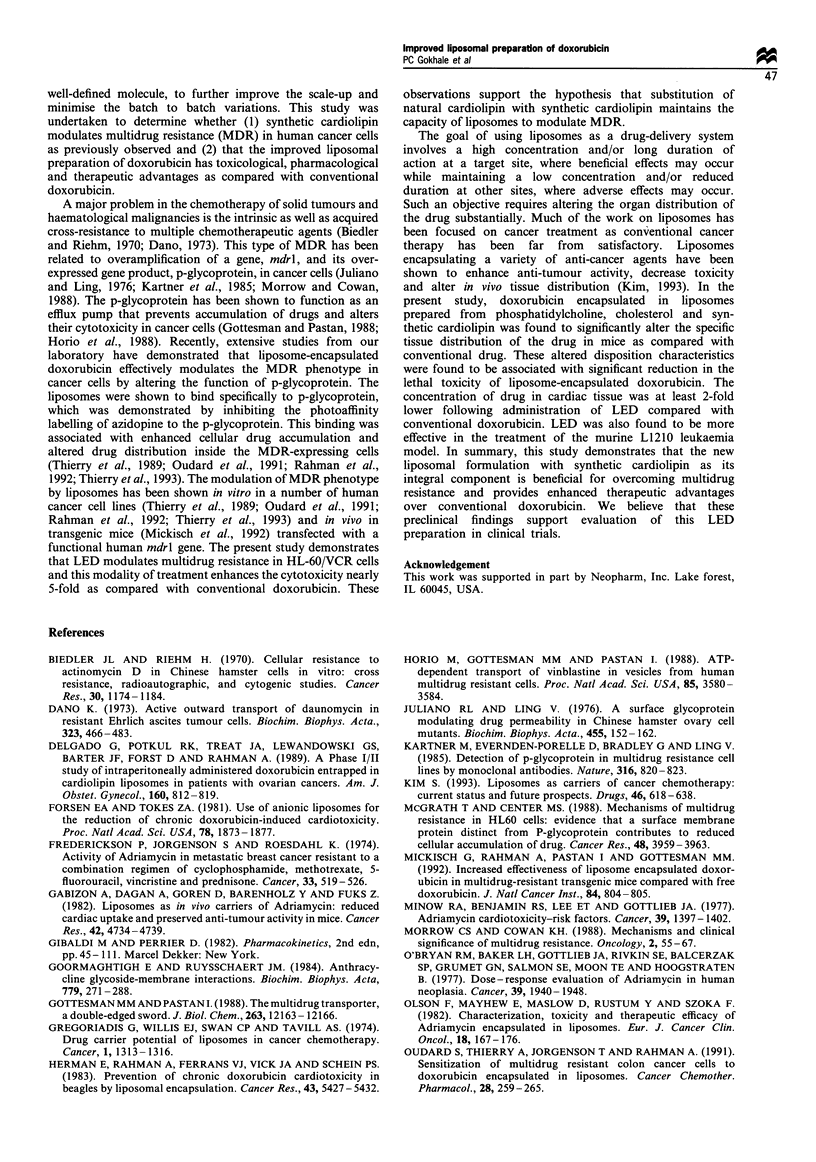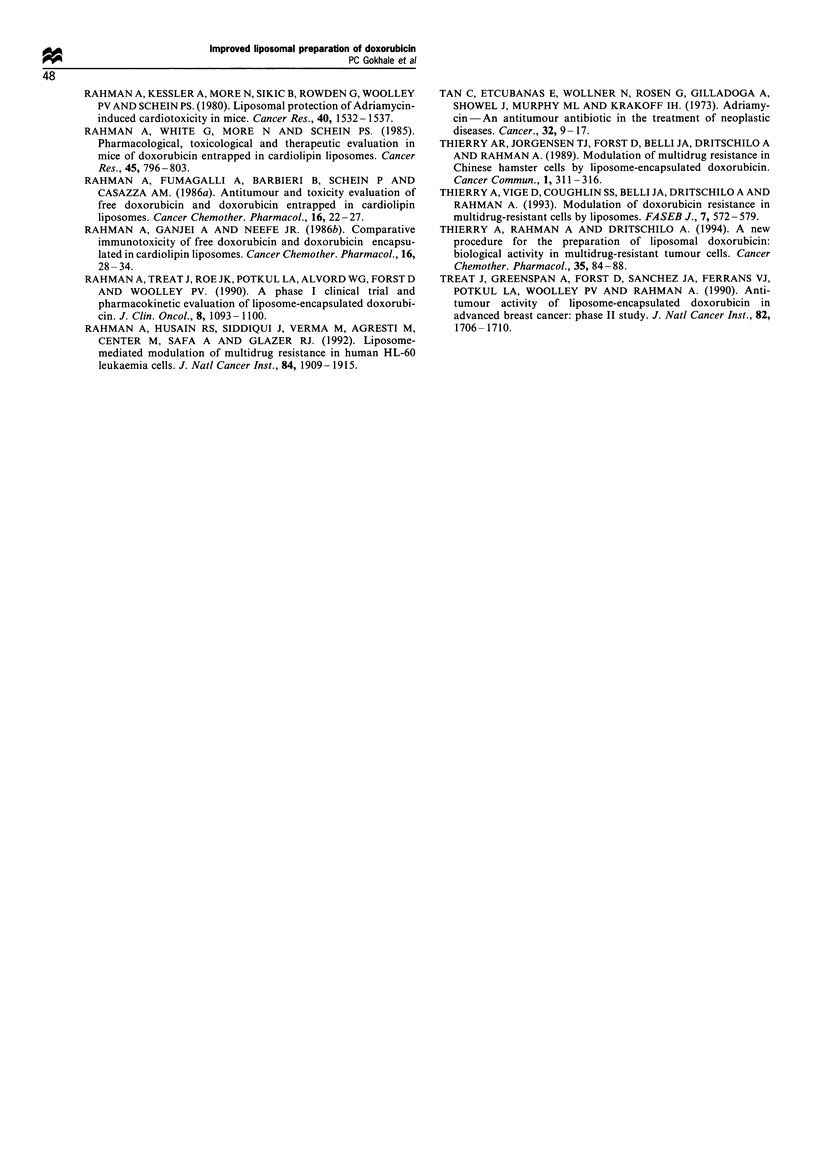# An improved method of encapsulation of doxorubicin in liposomes: pharmacological, toxicological and therapeutic evaluation.

**DOI:** 10.1038/bjc.1996.313

**Published:** 1996-07

**Authors:** P. C. Gokhale, B. Radhakrishnan, S. R. Husain, D. R. Abernethy, R. Sacher, A. Dritschilo, A. Rahman

**Affiliations:** Department of Radiology, Georgetown University Medical Center, Washington, DC 20007, USA.

## Abstract

We describe here an improved method of encapsulating doxorubicin in liposomes using phosphatidylcholine, cholesterol and synthetic tetramyristoyl cardiolipin. With this new composition of lipids the entrapment of doxorubicin was found to be > 90%. Cytotoxicity studies using vincristine-resistant HL-60/VCR leukaemia cells showed that liposome-encapsulated doxorubicin reverses multidrug resistance 5-fold compared with conventional doxorubicin and at levels equivalent to that obtained using liposomes with natural cardiolipin. In normal mice, liposome-encapsulated doxorubicin was much less toxic than the conventional drug. A dose of 25 mg kg-1 i.v. of conventional doxorubicin produced 100% mortality in mice by day 14, whereas liposomal doxorubicin exhibited only 10% mortality by day 60. Liposomal doxorubicin demonstrated enhanced anti-tumour activity against murine ascitic L1210 leukaemia compared with conventional doxorubicin. At a dose of 15 mg kg-1, liposomal doxorubicin increased the median life span with 12 of 18 long-term (60 days) survivors compared with only 3 of 18 with conventional drug. Mice injected i.v. with liposomal doxorubicin had plasma levels 44-fold higher than conventional doxorubicin, producing significantly higher (P < 0.02) area under the plasma concentration curve. An altered tissue distribution was also observed with liposomal doxorubicin; cardiac tissue demonstrating at least 2-fold lower levels with liposomal doxorubicin probably accounting for its lower toxicity. This altered pharmacokinetics of liposome-encapsulated doxorubicin, providing enhanced therapeutic advantage and the ability to modulate multidrug resistance, could be useful in a clinical setting.


British Journal of Cancer (1996) 74, 43-48
© 1996 Stockton Press All rights reserved 0007-0920/96 $12.00

### An improved method of encapsulation of doxorubicin in liposomes: pharmacological, toxicological and therapeutic evaluation

PC Gokhale[1], B Radhakrishnan[1], SR Husain[1], DR Abernethy[2], R Sacher[1], A Dritschilo[3] and A Rahman[1]

[1]*Department of Radiology and Laboratory Medicine,* [2]*Division of Clinical Pharmacology,* [3]*Department of Radiation Medicine, Georgetown University Medical Center, Washington, DC 20007, USA.*

**Summary** We describe here an improved method of encapsulating doxorubicin in liposomes using phosphatidylcholine, cholesterol and synthetic tetramyristoyl cardiolipin. With this new composition of lipids the entrapment of doxorubicin was found to be >90%. Cytotoxicity studies using vincristine-resistant HL-60/VCR leukaemia cells showed that liposome-encapsulated doxorubicin reverses multidrug resistance 5-fold compared with conventional doxorubicin and at levels equivalent to that obtained using liposomes with natural cardiolipin. In normal mice, liposome-encapsulated doxorubicin was much less toxic than the conventional drug. A dose of 25 mg $kg^{-1}$ i.v. of conventional doxorubicin produced 100% mortality in mice by day 14, whereas liposomal doxorubicin exhibited only 10% mortality by day 60. Liposomal doxorubicin demonstrated enhanced anti-tumour activity against murine ascitic L1210 leukaemia compared with conventional doxorubicin. At a dose of 15 mg $kg^{-1}$, liposomal doxorubicin increased the median life span with 12 of 18 long-term (60 days) survivors compared with only 3 of 18 with conventional drug. Mice injected i.v. with liposomal doxorubicin had plasma levels 44-fold higher than conventional doxorubicin, producing significantly higher ($P<0.02$) area under the plasma concentration curve. An altered tissue distribution was also observed with liposomal doxorubicin; cardiac tissue demonstrating at least 2-fold lower levels with liposomal doxorubicin probably accounting for its lower toxicity. This altered pharmacokinetics of liposome-encapsulated doxorubicin, providing enhanced therapeutic advantage and the ability to modulate multidrug resistance, could be useful in a clinical setting.

**Keywords:** liposome; doxorubicin; cardiolipin; multidrug resistance reversal; therapeutic efficacy

The anthracycline antibiotics play a prominent role in the treatment of leukaemia and solid tumours in humans. Among this class of compounds, doxorubicin has shown significant activity against a wide range of human cancers, including leukaemia, lymphomas and a variety of solid tumours (Tan *et al.*, 1973; Frederickson *et al.*, 1974). Unfortunately, the clinical use of the agent is limited by an unusual cardiomyopathy, which is related to the total cumulative dose of the drug (Minow *et al.*, 1977). Doxorubicin also produces acute toxicity in the form of bone marrow depression, alopecia and oral ulceration (O'Bryan *et al.*, 1977).

New formulations of doxorubicin using liposomes as delivery systems have been investigated to avoid treatment-limiting side-effects and to achieve better therapeutic efficacy (Forsen and Tokes, 1981; Gabizon *et al.*, 1982; Gregoriadis *et al.*, 1974; Olson *et al.*, 1982; Rahman *et al.*, 1980). Previously, our laboratory showed that encapsulation of doxorubicin into liposomes prepared from cardiolipin, egg phosphatidylcholine, cholesterol and stearylamine was less toxic than conventional doxorubicin in mice and beagle dogs (Herman *et al.*, 1983; Rahman *et al.*, 1985). At the same time, liposome-encapsulated doxorubicin (LED) had equivalent or greater anti-tumour activity than conventional doxorubicin against various murine tumour models (Rahman *et al.*, 1986*a*). Subsequently, LED was used for the phase I and phase II clinical trials (Rahman *et al.*, 1990; Treat *et al.*, 1990). LED was found to be well tolerated and demonstrated significant anti-tumour activity in patients with recurrent breast cancer (Treat *et al.*, 1990). Intraperitoneally administered LED was also found to be safe and effective in patients with ovarian cancer (Delgado *et al.*, 1989).

Correspondence: A Rahman, Georgetown University Medical Center, Department of Radiology, Preclinical Science Building, Room GD-9, 3900 Reservoir Road, Washington, DC 20007, USA
Received 22 June 1995; revised 30 October 1995; accepted 7 December 1995

Despite the encouraging clinical results observed in patients, further studies with LED could not be carried out primarily because of the problems related to the successful scale-up of the liposomes. Also the entrapment of doxorubicin into these liposomes was low (45–55%), requiring time-consuming processes to remove the unentrapped doxorubicin. Therefore, in order to improve the entrapment efficiency, we changed the liposome composition as well as substituting natural cardiolipin with synthetic tetramyristoyl cardiolipin. As the chemical composition, charge, structure and mode of preparation are all known variables that can modify the physicochemical, biological and pharmacological properties of the liposomes and therefore of the encapsulated drug, the present study was undertaken to evaluate the new liposomal doxorubicin formulation for its modulation of multidrug resistance, safety, efficacy and pharmacokinetic behaviour in animals.

### Materials and methods

### Chemicals

Doxorubicin was purchased from Adria Laboratories (Columbus, OH, USA) and egg phosphatidylcholine and cholesterol from Avanti Polar Lipids (Alabaster, AL, USA). 1,1',2,2'-Tetramyristoyl cardiolipin, synthesised by Avanti Polar Lipids was a generous gift. All other chemicals were reagent grade.

### Animals

Male CD2F1 mice and female DBA/2 mice, 8–10 weeks old, 20–25 g were purchased from the National Cancer Institute (Frederick, MD, USA). Mice were maintained according to accredited procedures in our facility, and fed purina chow and water *ad libitum*.

*Cell cultures*

The murine ascitic L1210 cell line was obtained from the Animal Genetics Branch, Developmental Therapeutics Program, Division of Cancer Treatment, National Cancer Institute, Frederick, MD, USA. The L1210 leukaemia was maintained by serial intraperitoneal (i.p.) passage in female DBA/2 mice.

HL-60 promyelocytic leukaemic cells were obtained from the American Type Culture Collection (Rockville, MD, USA). The vincristine-resistant subline HL-60/VCR (obtained from Dr Melvin Center), which expresses the multidrug resistance phenotype was maintained in suspension culture with 2 $\mu$M vincristine (McGrath and Center, 1988). Cultures were grown as suspensions in RPMI-1640 medium (Gibco Laboratories, NY, USA) supplemented with 10% heat-inactivated fetal bovine serum, 2 mM glutamine and penicillin/streptomycin. Cell lines were maintained at 37°C under 95% relative humidity in an atmosphere containing 5% carbon dioxide. The vincristine-resistant subline was maintained in drug-free medium for at least 1 week before study.

*Preparation of liposomes*

1,1',2,2'-Tetramyristoyl cardiolipin, phosphatidylcholine and cholesterol in a molar ratio of 1:10:6.8 were dissolved in chloroform–methanol (2:1) mixture and dried in a rotating flask under vacuum. Doxorubicin (1 mg $ml^{-1}$), in sterile normal saline was added to the dried lipid film. The drug to lipid molar ratio was 1:10. The film was hydrated and dispersed by vigorous vortexing. The hydrated suspension was sonicated for 30 min in a bath type sonicator (Heat Systems, W380) to yield small unilamellar liposomes. In separate experiments, lipids were lyophilised and doxorubicin in saline was added. The mixture was allowed to hydrate for 1 h and then sonicated to obtain small unilamellar liposomes. The unincorporated doxorubicin was separated from liposomally entrapped drug by extensive dialysis against sterile normal saline at 4°C over a period of 20 h or by gel chromatography using Sephadex G-50. The amount of doxorubicin encapsulated in liposomes was determined by fluorescence measurement (470 nm, excitation; 580 nm, emission). In all the experiments, the concentration of doxorubicin encapsulated in liposome was equivalent to the concentration of free drug used. The size of the liposomes as determined by flow cytometry was found to be less than 0.5 $\mu$m. Blank liposomes were prepared using the same composition of lipids in the absence of doxorubicin; they were diluted in sterile saline to yield lipid doses equivalent to those of liposome-encapsulated doxorubicin.

*Cytotoxicity assay*

The growth-inhibition method was used to determine the cytotoxicity of conventional doxorubicin and LED in human promyelocytic leukaemia cells. In brief, $5 \times 10^4$ HL-60 and HL-60/VCR cells were plated in tissue culture cell wells (well diameter 35 mm, Corning, NY, USA). Cells in exponential growth were exposed to varying concentrations of doxorubicin or LED for 72 h at 37°C. Cells were then counted in a haemocytometer, and viability was determined by trypan blue dye exclusion. The data is expressed in terms of $IC_{50}$, which corresponds to the concentration of the drug resulting in 50% survival of the cells compared with control.

*Toxicity evaluation*

For the comparison of lethal toxicity of conventional doxorubicin and LED, normal CD2F1 male mice received single i.v. doses of 15, 20 and 25 mg $kg^{-1}$. The mice were observed until day 60 after drug administration. The control mice received either saline or blank liposomes representing the same concentration of the lipid as was used to entrap a dose of 25.0 mg $kg^{-1}$ doxorubicin. Mice in each group were weighed and observed daily.

In vivo *antitumour activity study*

For *in vivo* anti-tumour studies, male CD2F1 mice were injected i.p. on day 0 with $1 \times 10^5$ viable L1210 leukaemia cells. On day 1, they were randomly divided into groups of ten mice. Each group received varying doses of either conventional doxorubicin or LED administered i.p. Control groups received saline or blank liposomes i.p. The injection volume was 2% body weight (0.2 ml 10 $g^{-1}$ body weight). Survival was recorded every 24 h and the mice were evaluated until day 60.

*Pharmacological disposition studies*

For pharmacological studies, male CD2F1 mice were injected i.v. via the tail vein with 6 mg $kg^{-1}$ of either conventional doxorubicin or LED. At 5 min, 15 min, 1 h, 2 h, 4 h, 8 h and 24 h after drug administration, four mice in each group were bled from the retro-orbital sinus into heparinised tubes and were killed by cervical dislocation. The blood was centrifuged at 2000 r.p.m. for 10 min at 4°C to separate the plasma. The liver, spleen, kidney, lung, heart and small intestine were rapidly excised and rinsed in ice-cold normal saline. The organs and plasma were frozen at −20°C until assayed for doxorubicin.

Plasma and tissue samples were analysed for doxorubicin by the method as previously described (Rahman *et al.*, 1986*b*). Plasma (0.25 ml) was diluted to 1 ml with distilled water, followed by addition of 0.2 ml of silver nitrate (33%, w/v). Tissues were homogenised in 1 ml of distilled water in a Polytron homogeniser (Brinkmann Instruments, Westbury, NY, USA) followed by addition of 0.2 ml of silver nitrate. The tubes were vortexed and 3 ml of *n*-butyl alcohol saturated with water was added. Each tube was vortexed vigorously for 1 min and then centrifuged at 5000 r.p.m. for 10 min. The organic layer was removed followed by further extraction of residue with 2 ml of *n*-butyl alcohol. The two extracts were pooled and doxorubicin was assayed spectrofluorometrically at 470 nm excitation and 580 nm emission. Control plasma and tissue samples obtained from mice treated with normal saline or blank liposomes were processed similarly to correct for any endogenous fluorescence. Standards were prepared by spiking known concentrations of doxorubicin in blank plasma and tissue to calculate the concentration of doxorubicin in samples.

Plasma pharmacokinetic parameters were assessed by standard methods (Gibaldi and Perrier, 1982). The elimination rate constant ($\beta$) was calculated from the linear regression analysis of plasma concentration *vs* time curve. The area under the curve ($AUC_{0\rightarrow\infty}$) was calculated using the linear trapezoidal method with extrapolation of the terminal phase to infinity ($C_{last}/\beta$), where $C_{last}$ is the last measured concentration. Other parameters calculated were as follows: Total body clearance (Cl) = Dose/AUC; volume of distribution ($V_{area}$) = Cl/$\beta$; elimination half-life ($t_{1/2\beta}$) = 0.693/$\beta$.

*Statistical methods*

The statistical significance of difference between means was calculated by Student's *t*-test. The Wilcoxon rank-sum test was used to compare group median survival. Differences in end point survival between conventional doxorubicin and LED was analysed by chi-square test. A $P < 0.05$ was considered to be statistically significant.

### Results

*Encapsulation efficiency of liposomes*

The encapsulation of doxorubicin into liposomes prepared using phosphatidylcholine, cholesterol and 1,1',2,2'-tetramyristoyl cardiolipin by conventional methods, was found to be greater than 90% ($n=20$). In other experiments, lipids were lyophilised and then reconstituted with doxorubicin solution in saline. In all these experiments, more than 90% encapsulation of doxorubicin was also observed ($n=20$).

### In vitro *cytotoxicity*

The concentration of conventional doxorubicin that caused 50% growth inhibition ($IC_{50}$) in the parent HL-60 cells after 3 days of exposure was 10.5 ng $ml^{-1}$, whereas it was 0.412 $\mu g\ ml^{-1}$ in HL-60/VCR cells. Hence, HL-60/VCR cells with multidrug resistance phenotype were 39-fold resistant to doxorubicin. When liposomal doxorubicin was used, the $IC_{50}$ for HL-60 cells was 7.5 ng $ml^{-1}$, which was similar to conventional doxorubicin. However, liposomal doxorubicin showed a 4.7-fold potentiation in cytotoxicity to HL-60/VCR cells compared with conventional doxorubicin ($P<0.001$); the $IC_{50}$ being 0.088 $\mu g\ ml^{-1}$ (Figure 1). Blank liposomes had no cytotoxic effects on either HL-60 or HL-60/VCR cells.

### In vivo *toxicity*

Various groups of normal CD2F1 mice received single i.v. injections of either conventional doxorubicin or LED at doses of 25.0, 20.0 and 15.0 mg $kg^{-1}$. Animals receiving conventional doxorubicin at a dose of 25.0 mg $kg^{-1}$ exhibited 100% mortality by day 13 (Table I). However, animals receiving a 25.0 mg $kg^{-1}$ dose of liposomal doxorubicin demonstrated only 10% mortality, with 90% of the animals surviving until the end of the study. At a dose of 20.0 mg $kg^{-1}$, 60% mortality was observed by day 27 with conventional doxorubicin, whereas no mortality was observed in the group of animals receiving the same dose of liposomal doxorubicin. Conventional doxorubicin at a dose of 15.0 mg $kg^{-1}$ produced 20% mortality in mice by day 60, while no mortality was observed with the same dose of drug entrapped in liposomes.

Variation in body weight was recorded in all groups of mice treated with either conventional drug or liposomal doxorubicin. The mice treated with saline or blank liposomes had a progressive increase in body weight. Mice treated with 25 mg $kg^{-1}$ conventional doxorubicin had a rapid weight loss, amounting to 19% by day 6. Mice that received 25.0 mg $kg^{-1}$ LED had a maximum weight loss of 14% until day 10; with a progressive weight gain after day 10 (data not shown).

### In vivo *activity*

To determine the anti-tumour activity of doxorubicin entrapped in liposomes, studies were carried out against the murine L1210 ascitic leukaemia in CD2F1 mice (Figure 2). Twenty-four hours after i.p. implantation of $1\times10^5$ L1210 tumour cells, mice were given i.p. injections of conventional doxorubicin or doxorubicin entrapped in liposomes in varying doses. A dose of 15 mg $kg^{-1}$ was most effective with both types of treatment. The median survival time with conventional doxorubicin was 16.5 days and there were 3 of 18 long-term survivors. Treatment with LED significantly increased the anti-tumour activity ($P<0.001$) and there were 12 of 18 long-term survivors.

**Table I** Toxicity evaluation of conventional doxorubicin and liposome encapsulated doxorubicin in CD2F1 mice

| *Dose (mg $kg^{-1}$)* | *Median survival (days)* | | *Sixty day survival(%)* | |
|---|---|---|---|---|
| | *Dox* | *LED* | *Dox* | *LED* |
| 15.0 | >60 | >60 | 80 | 100* |
| 20.0 | 25.5 (10–60) | >60 | 40 | 100* |
| 25.0 | 7.0 (6–13) | >60 | 0 | 90* |

CD2F1 mice received single i.v. injection of conventional doxorubicin (Dox) or liposome-encapsulated doxorubicin (LED). At each dose level groups consisted of ten animals. The numbers in parenthesis represents range. *$P<0.001$.

**Figure 1** Cytotoxicity of doxorubicin and liposome-encapsulated doxorubicin in HL-60 (**a**) and HL-60/VCR cells (**b**). Cells were exposed with conventional doxorubicin (○) or doxorubicin encapsulated in liposomes (●) for 72 h and cytotoxicity was determined by growth inhibition assays as described in Materials and methods. Each value is the mean±s.d. of three experiments carried out in duplicate.

**Figure 2** Anti-tumour efficacy of conventional doxorubicin (□) and doxorubicin encapsulated in liposomes (▨) against L1210 murine leukaemia in CD2F1 mice. Mice were inoculated i.p. with $1\times10^5$ L1210 cells and treated with varying doses of drug administered i.p. 24 h after tumour implantation. Values over each bar (alive/number of animals treated) represent long-term survival (60 days). The median survival time of control group treated with either saline or blank liposomes ($n=24$) was 7.5 days. Also shown is the interquartile range, indicated by error bars, between the 25th percentile and the 75th percentile. *$P<0.001$.

*Pharmacokinetics*

The plasma pharmacokinetics of conventional doxorubicin and liposome-encapsulated doxorubicin in mice at a dose of 6 mg $kg^{-1}$ is presented in Figure 3. Following i.v. administration of conventional doxorubicin, the peak plasma concentration achieved was 0.867 $\mu g\ ml^{-1}$. On the other hand, with liposomes, the peak plasma concentration of doxorubicin achieved was 38.04 $\mu g\ ml^{-1}$, which was 43.8 times more than conventional doxorubicin. The comparative pharmacokinetic parameters obtained after a single i.v. bolus administration of conventional doxorubicin and doxorubicin entrapped in liposomes are presented in Table II. LED produced significantly higher ($P<0.02$) area under the plasma–concentration–time curve compared with conventional doxorubicin. The calculated volume of distribution at steady state was substantially reduced with LED as compared with conventional doxorubicin, and calculated total doxorubicin clearance was also markedly reduced ($P<0.01$).

**Figure 3** Plasma concentration–time relationship for conventional doxorubicin (○) and doxorubicin encapsulated in liposomes (●). Male CD2F1 mice were injected i.v. with a 6 mg $kg^{-1}$ dose of conventional doxorubicin or liposome-encapsulated doxorubicin and plasma levels of doxorubicin equivalents were determined as described in Materials and methods. Each point represents a mean ± s.d. of four animals.

The tissue distribution of conventional doxorubicin and doxorubicin entrapped in liposomes is presented in Table III. The peak cardiac concentration with conventional doxorubicin was 16.9 $\mu g\ g^{-1}$ at 15 min, whereas it was 7.7 $\mu g\ g^{-1}$ at 5 min with liposomal doxorubicin. The cardiac tissue concentration of doxorubicin after administration of LED was at least 2-fold less ($P<0.001$) than conventional drug, and this relationship was maintained over 8 h. The liver and spleen demonstrated significantly higher ($P<0.001$) accumulation of LED compared with conventional doxorubicin. Drug levels in the small intestine showed a modest decrease with LED, but no significant difference was seen in the area under the curve.

### Discussion

This report describes a new method of preparation of liposome-encapsulated doxorubicin with greater simplicity that makes the scale-up of these liposomes much easier. Cardiolipin, a diphosphatidylglycerol containing two negative charges, has been shown to have a high affinity for doxorubicin, which contains one positive charge. In our previous study with liposome-encapsulated doxorubicin (Rahman *et al*., 1985), the liposomes were composed of cardiolipin, phosphatidylcholine, cholesterol and a positively charged stearylamine. In these studies, the low entrapment of doxorubicin was because of interaction of stearylamine with cardiolipin. Elimination of stearylamine from the liposomal preparation mixture was associated with increased doxorubicin entrapment. With this new formulation the entrapment of doxorubicin was found to be >90%. In separate experiments, we found that by simply mixing and vortexing conventional drug with lyophilised lipids, similar level of encapsulation could be achieved. This procedure of preparing liposomes can simplify the industrial manufacturing process and make this drug available to patients for treatment. As shown previously (Thierry *et al*., 1994), the complex formation between doxorubicin and cardiolipin is strongly stabilised by an electrostatic interaction between two molecules of doxorubicin and one molecule of cardiolipin and a stoichiometric interaction which leads to a card-pack dimer formation (Goormaghtigh and Ruysschaert, 1984). In this study, we have replaced natural cardiolipin, which has a heterogenous fatty acid structure with synthetic cardiolipin, a

**Table II** Pharmacokinetic parameters of conventional doxorubicin and liposomal doxorubicin in CD2F1 mice after single i.v. dose of 6 mg $kg^{-1}$

| | $t_{\frac{1}{2}\beta}$ (h) | $AUC_{0\rightarrow\infty}$ ($\mu g\ h\ ml^{-1}$) | $Cl$ ($ml\ h^{-1}\ kg^{-1}$) | $V_{area}$ ($l\ kg^{-1}$) |
|---|---|---|---|---|
| Conventional doxorubicin | 12.6 ± 0.4* | 3.19 ± 0.5* | 1902.4 ± 286.7** | 34.7 ± 6.4** |
| Liposomal doxorubicin | 8.8 ± 0.8 | 32.5 ± 6.9 | 188.6 ± 39.6 | 2.4 ± 0.7 |

Values are means ± s.d. $t_{\frac{1}{2}\beta}$, elimination half-life; AUC, area under the plasma-concentration–time curve; Cl, total body clearance; $V_{area}$, volume of distribution; *$P<0.02$, **$P<0.01$.

**Table III** Tissue distribution of doxorubicin after i.v. administration of 6 mg $kg^{-1}$ of conventional doxorubicin or liposome-encapsulated doxorubicin

| | *Heart* | | *Liver* | | *Spleen* | | *Lungs* | | *Kidneys* | | *Small intestine* | |
|---|---|---|---|---|---|---|---|---|---|---|---|---|
| *Time* | *Dox* | *LED* | *Dox* | *LED* | *Dox* | *LED* | *Dox* | *LED* | *Dox* | *LED* | *Dox* | *LED* |
| 5 min | 14.8 ± 1.8 | 7.7 ± 0.9 | 54.8 ± 2.6 | 47.2 ± 2.7 | 19.2 ± 5.3 | 58.7 ± 8.5 | 24.1 ± 3.4 | 8.6 ± 0.9 | 36.1 ± 6.1 | 12.2 ± 0.7 | 4.5 ± 2.5 | 1.1 ± 0.2 |
| 15 min | 16.9 ± 0.5 | 7.2 ± 1.2 | 57.0 ± 4.8 | 56.3 ± 4.8 | 14.1 ± 3.7 | 85.4 ± 5.9 | 20.3 ± 1.6 | 11.5 ± 1.7 | 28.9 ± 2.0 | 11.8 ± 0.5 | 5.3 ± 1.9 | 1.3 ± 0.3 |
| 1 h | 10.9 ± 2.4 | 4.9 ± 0.7 | 25.1 ± 3.1 | 53.9 ± 11.3 | 13.4 ± 1.2 | 139.6 ± 19.6 | 19.6 ± 2.6 | 6.0 ± 1.5 | 22.7 ± 2.5 | 11.4 ± 2.5 | 1.2 ± 0.7 | 0.7 ± 0.2 |
| 2 h | 9.5 ± 0.9 | 3.2 ± 0.6 | 27.1 ± 4.8 | 57.7 ± 6.6 | 13.5 ± 0.8 | 117.5 ± 22.6 | 14.5 ± 3.4 | 5.2 ± 0.7 | 25.5 ± 5.2 | 10.2 ± 1.1 | 1.4 ± 0.3 | 0.8 ± 0.4 |
| 4 h | 6.4 ± 0.5 | 2.8 ± 0.2 | 24.7 ± 3.0 | 51.0 ± 4.9 | 13.7 ± 1.4 | 156.7 ± 18.7 | 14.1 ± 0.2 | 5.0 ± 0.4 | 21.0 ± 3.8 | 10.5 ± 0.9 | 1.1 ± 0.8 | 0.7 ± 0.2 |
| 8 h | 3.9 ± 0.4 | 1.9 ± 0.1 | 15.7 ± 1.8 | 51.5 ± 2.8 | 17.1 ± 1.6 | 172.1 ± 15.3 | 10.2 ± 1.2 | 4.2 ± 0.5 | 17.6 ± 0.8 | 8.6 ± 1.5 | 0.6 ± 0.3 | 0.6 ± 0.2 |
| 24 h | 1.5 ± 0.1 | 1.3 ± 0.1 | 3.9 ± 0.5 | 18.3 ± 2.5 | 13.9 ± 1.1 | 179.5 ± 16.3 | 6.3 ± 1.1 | 6.5 ± 0.5 | 6.0 ± 0.6 | 5.0 ± 0.9 | 0.4 ± 0.1 | 0.2 ± 0.1 |
| $AUC_0^{24}$ ($\mu g\ h\ g^{-1}$) | 103.6 ± 10.4 | 52.1* ± 4.4 | 356.3 ± 43.4 | 979.4* ± 80.4 | 364.0 ± 32.9 | 3980.0* ± 372.8 | 245.8 ± 29.1 | 128.7* ± 13.4 | 371.8 ± 33.4 | 191.0* ± 27.7 | 18.9 ± 8.2 | 12.6 ± 4.3 |

Values are means ± s.d. of four mice ($\mu g\ g^{-1}$ tissue). Dox, conventional doxorubicin; LED, liposome-encapsulated doxorubicin; AUC, area under the curve. *$P<0.001$.

well-defined molecule, to further improve the scale-up and minimise the batch to batch variations. This study was undertaken to determine whether (1) synthetic cardiolipin modulates multidrug resistance (MDR) in human cancer cells as previously observed and (2) that the improved liposomal preparation of doxorubicin has toxicological, pharmacological and therapeutic advantages as compared with conventional doxorubicin.

A major problem in the chemotherapy of solid tumours and haematological malignancies is the intrinsic as well as acquired cross-resistance to multiple chemotherapeutic agents (Biedler and Riehm, 1970; Dano, 1973). This type of MDR has been related to overamplification of a gene, *mdr*1, and its over-expressed gene product, p-glycoprotein, in cancer cells (Juliano and Ling, 1976; Kartner *et al.*, 1985; Morrow and Cowan, 1988). The p-glycoprotein has been shown to function as an efflux pump that prevents accumulation of drugs and alters their cytotoxicity in cancer cells (Gottesman and Pastan, 1988; Horio *et al.*, 1988). Recently, extensive studies from our laboratory have demonstrated that liposome-encapsulated doxorubicin effectively modulates the MDR phenotype in cancer cells by altering the function of p-glycoprotein. The liposomes were shown to bind specifically to p-glycoprotein, which was demonstrated by inhibiting the photoaffinity labelling of azidopine to the p-glycoprotein. This binding was associated with enhanced cellular drug accumulation and altered drug distribution inside the MDR-expressing cells (Thierry *et al.*, 1989; Oudard *et al.*, 1991; Rahman *et al.*, 1992; Thierry *et al.*, 1993). The modulation of MDR phenotype by liposomes has been shown *in vitro* in a number of human cancer cell lines (Thierry *et al.*, 1989; Oudard *et al.*, 1991; Rahman *et al.*, 1992; Thierry *et al.*, 1993) and *in vivo* in transgenic mice (Mickisch *et al.*, 1992) transfected with a functional human *mdr*1 gene. The present study demonstrates that LED modulates multidrug resistance in HL-60/VCR cells and this modality of treatment enhances the cytotoxicity nearly 5-fold as compared with conventional doxorubicin. These observations support the hypothesis that substitution of natural cardiolipin with synthetic cardiolipin maintains the capacity of liposomes to modulate MDR.

The goal of using liposomes as a drug-delivery system involves a high concentration and/or long duration of action at a target site, where beneficial effects may occur while maintaining a low concentration and/or reduced duration at other sites, where adverse effects may occur. Such an objective requires altering the organ distribution of the drug substantially. Much of the work on liposomes has been focused on cancer treatment as conventional cancer therapy has been far from satisfactory. Liposomes encapsulating a variety of anti-cancer agents have been shown to enhance anti-tumour activity, decrease toxicity and alter *in vivo* tissue distribution (Kim, 1993). In the present study, doxorubicin encapsulated in liposomes prepared from phosphatidylcholine, cholesterol and synthetic cardiolipin was found to significantly alter the specific tissue distribution of the drug in mice as compared with conventional drug. These altered disposition characteristics were found to be associated with significant reduction in the lethal toxicity of liposome-encapsulated doxorubicin. The concentration of drug in cardiac tissue was at least 2-fold lower following administration of LED compared with conventional doxorubicin. LED was also found to be more effective in the treatment of the murine L1210 leukaemia model. In summary, this study demonstrates that the new liposomal formulation with synthetic cardiolipin as its integral component is beneficial for overcoming multidrug resistance and provides enhanced therapeutic advantages over conventional doxorubicin. We believe that these preclinical findings support evaluation of this LED preparation in clinical trials.

**Acknowledgement**
This work was supported in part by Neopharm, Inc. Lake forest, IL 60045, USA.

### References

BIEDLER JL AND RIEHM H. (1970). Cellular resistance to actinomycin D in Chinese hamster cells in vitro: cross resistance, radioautographic, and cytogenic studies. *Cancer Res.*, **30,** 1174–1184.

DANO K. (1973). Active outward transport of daunomycin in resistant Ehrlich ascites tumour cells. *Biochim. Biophys. Acta.*, **323,** 466–483.

DELGADO G, POTKUL RK, TREAT JA, LEWANDOWSKI GS, BARTER JF, FORST D AND RAHMAN A. (1989). A Phase I/II study of intraperitoneally administered doxorubicin entrapped in cardiolipin liposomes in patients with ovarian cancers. *Am. J. Obstet. Gynecol.*, **160,** 812–819.

FORSEN EA AND TOKES ZA. (1981). Use of anionic liposomes for the reduction of chronic doxorubicin-induced cardiotoxicity. *Proc. Natl Acad. Sci. USA*, **78,** 1873–1877.

FREDERICKSON P, JORGENSON S AND ROESDAHL K. (1974). Activity of Adriamycin in metastatic breast cancer resistant to a combination regimen of cyclophosphamide, methotrexate, 5-fluorouracil, vincristine and prednisone. *Cancer*, **33,** 519–526.

GABIZON A, DAGAN A, GOREN D, BARENHOLZ Y AND FUKS Z. (1982). Liposomes as *in vivo* carriers of Adriamycin: reduced cardiac uptake and preserved anti-tumour activity in mice. *Cancer Res.*, **42,** 4734–4739.

GIBALDI M AND PERRIER D. (1982). *Pharmacokinetics*, 2nd edn, pp. 45–111. Marcel Dekker: New York.

GOORMAGHTIGH E AND RUYSSCHAERT JM. (1984). Anthracycline glycoside-membrane interactions. *Biochim. Biophys. Acta*, **779,** 271–288.

GOTTESMAN MM AND PASTAN I. (1988). The multidrug transporter, a double-edged sword. *J. Biol. Chem.*, **263,** 12163–12166.

GREGORIADIS G, WILLIS EJ, SWAN CP AND TAVILL AS. (1974). Drug carrier potential of liposomes in cancer chemotherapy. *Cancer*, **1,** 1313–1316.

HERMAN E, RAHMAN A, FERRANS VJ, VICK JA AND SCHEIN PS. (1983). Prevention of chronic doxorubicin cardiotoxicity in beagles by liposomal encapsulation. *Cancer Res.*, **43,** 5427–5432.

HORIO M, GOTTESMAN MM AND PASTAN I. (1988). ATP-dependent transport of vinblastine in vesicles from human multidrug resistant cells. *Proc. Natl Acad. Sci. USA*, **85,** 3580–3584.

JULIANO RL AND LING V. (1976). A surface glycoprotein modulating drug permeability in Chinese hamster ovary cell mutants. *Biochim. Biophys. Acta.*, **455,** 152–162.

KARTNER M, EVERNDEN-PORELLE D, BRADLEY G AND LING V. (1985). Detection of p-glycoprotein in multidrug resistance cell lines by monoclonal antibodies. *Nature*, **316,** 820–823.

KIM S. (1993). Liposomes as carriers of cancer chemotherapy: current status and future prospects. *Drugs*, **46,** 618–638.

MCGRATH T AND CENTER MS. (1988). Mechanisms of multidrug resistance in HL60 cells: evidence that a surface membrane protein distinct from P-glycoprotein contributes to reduced cellular accumulation of drug. *Cancer Res.*, **48,** 3959–3963.

MICKISCH G, RAHMAN A, PASTAN I AND GOTTESMAN MM. (1992). Increased effectiveness of liposome encapsulated doxorubicin in multidrug-resistant transgenic mice compared with free doxorubicin. *J. Natl Cancer Inst.*, **84,** 804–805.

MINOW RA, BENJAMIN RS, LEE ET AND GOTTLIEB JA. (1977). Adriamycin cardiotoxicity–risk factors. *Cancer*, **39,** 1397–1402.

MORROW CS AND COWAN KH. (1988). Mechanisms and clinical significance of multidrug resistance. *Oncology*, **2,** 55–67.

O'BRYAN RM, BAKER LH, GOTTLIEB JA, RIVKIN SE, BALCERZAK SP, GRUMET GN, SALMON SE, MOON TE AND HOOGSTRATEN B. (1977). Dose–response evaluation of Adriamycin in human neoplasia. *Cancer*, **39,** 1940–1948.

OLSON F, MAYHEW E, MASLOW D, RUSTUM Y AND SZOKA F. (1982). Characterization, toxicity and therapeutic efficacy of Adriamycin encapsulated in liposomes. *Eur. J. Cancer Clin. Oncol.*, **18,** 167–176.

OUDARD S, THIERRY A, JORGENSON T AND RAHMAN A. (1991). Sensitization of multidrug resistant colon cancer cells to doxorubicin encapsulated in liposomes. *Cancer Chemother. Pharmacol.*, **28,** 259–265.

RAHMAN A, KESSLER A, MORE N, SIKIC B, ROWDEN G, WOOLLEY PV AND SCHEIN PS. (1980). Liposomal protection of Adriamycin-induced cardiotoxicity in mice. *Cancer Res.*, **40,** 1532–1537.

RAHMAN A, WHITE G, MORE N AND SCHEIN PS. (1985). Pharmacological, toxicological and therapeutic evaluation in mice of doxorubicin entrapped in cardiolipin liposomes. *Cancer Res.*, **45,** 796–803.

RAHMAN A, FUMAGALLI A, BARBIERI B, SCHEIN P AND CASAZZA AM. (1986*a*). Antitumour and toxicity evaluation of free doxorubicin and doxorubicin entrapped in cardiolipin liposomes. *Cancer Chemother. Pharmacol.*, **16,** 22–27.

RAHMAN A, GANJEI A AND NEEFE JR. (1986*b*). Comparative immunotoxicity of free doxorubicin and doxorubicin encapsulated in cardiolipin liposomes. *Cancer Chemother. Pharmacol.*, **16,** 28–34.

RAHMAN A, TREAT J, ROE JK, POTKUL LA, ALVORD WG, FORST D AND WOOLLEY PV. (1990). A phase I clinical trial and pharmacokinetic evaluation of liposome-encapsulated doxorubicin. *J. Clin. Oncol.*, **8,** 1093–1100.

RAHMAN A, HUSAIN RS, SIDDIQUI J, VERMA M, AGRESTI M, CENTER M, SAFA A AND GLAZER RJ. (1992). Liposome-mediated modulation of multidrug resistance in human HL-60 leukaemia cells. *J. Natl Cancer Inst.*, **84,** 1909–1915.

TAN C, ETCUBANAS E, WOLLNER N, ROSEN G, GILLADOGA A, SHOWEL J, MURPHY ML AND KRAKOFF IH. (1973). Adriamycin—An antitumour antibiotic in the treatment of neoplastic diseases. *Cancer.*, **32,** 9–17.

THIERRY AR, JORGENSEN TJ, FORST D, BELLI JA, DRITSCHILO A AND RAHMAN A. (1989). Modulation of multidrug resistance in Chinese hamster cells by liposome-encapsulated doxorubicin. *Cancer Commun.*, **1,** 311–316.

THIERRY A, VIGE D, COUGHLIN SS, BELLI JA, DRITSCHILO A AND RAHMAN A. (1993). Modulation of doxorubicin resistance in multidrug-resistant cells by liposomes. *FASEB J.*, **7,** 572–579.

THIERRY A, RAHMAN A AND DRITSCHILO A. (1994). A new procedure for the preparation of liposomal doxorubicin: biological activity in multidrug-resistant tumour cells. *Cancer Chemother. Pharmacol.*, **35,** 84–88.

TREAT J, GREENSPAN A, FORST D, SANCHEZ JA, FERRANS VJ, POTKUL LA, WOOLLEY PV AND RAHMAN A. (1990). Antitumour activity of liposome-encapsulated doxorubicin in advanced breast cancer: phase II study. *J. Natl Cancer Inst.*, **82,** 1706–1710.